# Thoracic abnormal air collections in patients in the intensive care unit: radiograph findings correlated with CT

**DOI:** 10.1186/s13244-020-0838-z

**Published:** 2020-03-12

**Authors:** Masafumi Sakai, Takashi Hiyama, Hirofumi Kuno, Kensaku Mori, Tsukasa Saida, Toshitaka Ishiguro, Hiroaki Takahashi, Ken Koyama, Manabu Minami

**Affiliations:** 1 Department of Diagnostic and Interventional Radiology, Ibaraki Prefectural Central Hospital, Kasama, Japan; 2 Department of Diagnostic and Interventional Radiology, University of Tsukuba Hospital, Amakubo 2-1-1, Tsukuba, Ibaraki, 305-8576 Japan; 3 Department of Diagnostic Radiology, National Cancer Center Hospital East, Kashiwa, Japan; 4 Department of Diagnostic Radiology, Mayo Clinic Minnesota, 200 First St. SW, Rochester, MN 55905 USA

**Keywords:** Radiograph, Computed tomography, Air collection, Thorax, Intensive care unit

## Abstract

An abnormal collection of air in the thorax is one of the most common life-threatening events that occurs in the intensive care unit. Patient management differs depending on the location of the air collection; therefore, detecting abnormal air collection and identifying its exact location on supine chest radiographs is essential for early treatment and positive patient outcomes. Thoracic abnormal air collects in multiple thoracic spaces, including the pleural cavity, chest wall, mediastinum, pericardium, and lung. Pneumothorax in the supine position shows different radiographic findings depending on the location. Many conditions, such as skin folds, interlobar fissure, bullae in the apices, and air collection in the intrathoracic extrapleural space, mimic pneumothorax on radiographs. Additionally, pneumopericardium may resemble pneumomediastinum and needs to be differentiated. Further, some conditions such as inferior pulmonary ligament air collection versus a pneumatocele or pneumothorax in the posteromedial space require a differential diagnosis based on radiographs. Computed tomography (CT) is required to localize the air and delineate potential etiologies when a diagnosis by radiography is difficult. The purposes of this article are to review the anatomy of the potential spaces in the chest where abnormal air can collect, explain characteristic radiographic findings of the abnormal air collection in supine patients with illustrations and correlated CT images, and describe the distinguishing features of conditions that require a differential diagnosis. Since management differs based on the location of the air collection, radiologists should try to accurately detect and identify the location of air collection on supine radiographs.

## Key points

- Management differs based on the location of thoracic abnormal air collection.
- Identifying abnormal air collection on radiographs is essential for early treatment.
- Air collection is classified into the pleural cavity, chest wall, etc.
- Radiographic findings vary depending on the location of the lesion.
- Understanding CT anatomy enables locating abnormal thoracic air on radiographs.

## Background

A portable chest radiograph is the most commonly used radiographic examination in the intensive care unit (ICU) [1]. Despite its diagnostic limitations [2, 3], chest radiographs often reveal critical abnormalities that may remain unnoticed clinically. One of the life-threatening events in the ICU is the development of abnormal air collection in the thorax caused by interstitial or cystic lung disease, infectious diseases, trauma, positive pressure ventilation, and other complications associated with medical interventions [4–11].

The management of abnormal air collections in the thorax depends on its location. Some abnormal air collections are managed through careful observation, and other air collections require surgical intervention. Pneumothorax, especially tension pneumothorax, is treated by drainage [12]. Subcutaneous emphysema, air collection in the intrathoracic extrapleural space, and pneumomediastinum are usually not considered urgent conditions and often only require clinical observation [13–15]. However, in the case of abnormal air collection caused by tracheal/esophageal rupture, emergent surgical intervention may be required. Pneumopericardium is treated by emergent pericardiocentesis in a case of cardiac tamponade [9]. The main treatment goal of pulmonary interstitial emphysema (PIE) is to achieve adequate oxygenation with a lower mean and peak airway pressure of ventilation [16]. Consequently, the detection and accurate localization of abnormal air collection is essential for early and optimal treatment and favorable patient outcomes.

Abnormal air collection can be more accurately evaluated by computed tomography (CT) than by chest radiography [3]. Nevertheless, CT is not easily conducted in an ICU setting because patients are critically ill and often supported by many devices. Lung ultrasound is readily available and may effectively and accurately detect pneumothorax as the absence of lung sliding and comet-tail artifact, but its outcomes are contingent on the operator’s skill. Additionally, it is often difficult to observe deeper regions of the chest and obtain a complete view [17]. Consequently, portable chest radiography with a patient in the supine position is the fundamental imaging examination that is conducted in the ICU. However, air collection can be easily overlooked on supine radiographs if a clinician does not have a comprehensive understanding of the three-dimensional anatomy of the chest and the predicted sites of abnormal air collection.

The purposes of this article are to review the anatomy of potential spaces in the chest where air can collect, explain the characteristic radiographic findings of abnormal air collection in supine patients using illustrations and correlated CT images, and describe the distinguishing features of conditions that require a differential diagnosis. We classify the chest into the following five locations based on its anatomy (Table 1): the pleural cavity, chest wall, mediastinum, pericardium, and lung. We also described diseases of abnormal air collections in each of these spaces (Table 1).

**Table 1:** Classification of thoracic abnormal air collection based on anatomy

Location	Anatomical structure and space	Disease of abnormal air collection
I. Pleural cavity	Anteromedial space	Pneumothorax in the anteromedial space (I-1)
	Subpulmonary space	Pneumothorax in the subpulmonary space (I-2)
	Apicolateral space	Pneumothorax in the apicolateral space (I-3)
	Posteromedial space	Pneumothorax in the posteromedial space (I-4)
	(Emergency condition)	Tension pneumothorax (I-5)
	(Differential diagnosis)	Mimics of pneumothorax (I-6)
II. Chest wall	Subcutaneous space	Subcutaneous emphysema (II-1)
	Intrathoracic extrapleural space	Air collection in the intrathoracic extrapleural space (II-2)
III. Mediastinum	Mediastinum	Pneumomediastinum (III-1)
	Inferior pulmonary ligament	Air collection in the inferior pulmonary ligament (III-2)
IV. Pericardium	Pericardium	Pneumopericardium (IV-1)
V. Lung	Interstitium	Pulmonary interstitial emphysema (V-1)
	Parenchyma	Pneumatocele (V-2)

The numbers in the parentheses correspond to the numbers in the text and figures

## Abnormal air collection in the pleural cavity

**Fig. 1:**
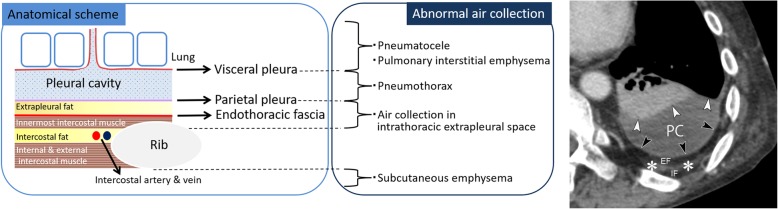
Anatomical scheme of the pleural cavity and the chest wall and disease of abnormal air collection in each anatomical space. Axial chest CT of a 50-year-old man with chronic empyema shows fluid collection in the pleural cavity and thickened pleura. The CT shows the visceral pleura (white arrowheads), pleural cavity (PC), parietal pleura (black arrowheads), extrapleural fat (EF), innermost intercostal muscle and endothoracic fascia (white asterisks), and intercostal fat (IF). The innermost intercostal muscle is normally defective in the dorsal mediastinal side of the chest wall

The pleural cavity and chest wall are anatomically classified into several layers (Fig. 1), and abnormal air predominantly collects in each anatomical space (Fig. 1). The pleural space is the area between the visceral and the parietal pleurae (Fig. 1). The visceral pleura covers the lung and folds back on itself at the root of the lung (pulmonary hilum) to become the parietal pleura (Fig. 1). The pleural space normally contains a small amount of fluid (between 1 and 5 mL) [18], and the pleural space is not visualized on radiographs or CT images without pleural effusion or pneumothorax. The pleural space is primarily classified as anteromedial, subpulmonary, apicolateral, and posteromedial [19, 20].

### Pneumothorax

Pneumothorax is a disease characterized by the collection of air in one or several spaces of the pleural cavity [21]. Here, we describe pneumothorax in each space; tension pneumothorax, which is an emergent condition; and conditions that require a differential diagnosis of pneumothorax. The main symptoms of pneumothorax are sudden chest pain, dyspnea, and dry cough. Pneumothorax is caused by the rupture of a bulla, trauma, and iatrogenic complications after an intervention, such as thoracentesis and positive pressure ventilation [4]. In the ICU, pneumothorax is commonly caused by barotrauma associated with ventilation [4]. Pneumothorax occurs in nearly 20% of patients with thoracic trauma [4]. It is treated with thoracic drainage. Based on the British Thoracic Society Guidelines 2010, a large pneumothorax is defined on erect chest radiographs as an air space that is more than 2 cm from the pleural surface to the lung edge at the level of the hilum [22]. A large pneumothorax usually requires thoracentesis, and emergency thoracentesis is necessary to treat tension pneumothorax [22]. Conversely, patients with a small pneumothorax and without significant breathlessness are usually managed through clinical observation [22]. Air mainly collects in the apicolateral space in erect patients. Saeki [23] reported that 62 of 73 (84.9%) pneumothoraces were detected in the apicolateral space on erect radiographs. A radiographic sign of pneumothorax in erect patients is characterized by the visualization of a thin visceral pleural line in the apicolateral space with no vascular markings beyond that line [19]. Since there are few overlapping structures in the apicolateral space, such as the mediastinum, pneumothorax can be easily identified in an erect radiograph. In supine patients, the spaces where air collects are different because air in the pleural cavity is influenced by gravity, lung recoil due to adhesion, and the anatomy of the pleural cavity [19, 24]. Tocino et al. [20] reported that the air collection space was anteromedial in 44%, subpulmonary in 29%, apicolateral in 13%, and posteromedial in 13% of 68 cases of pneumothorax in ICU supine patients [20].

### Pneumothorax in the anteromedial space (I-1)

In supine patients, the anteromedial space is the least dependent pleural space [19, 20, 24]. Therefore, pneumothorax in the anteromedial space is most commonly observed in supine patients [20]. The anteromedial space is divided by the hilum into the suprahilar space and the infrahilar space.

**Fig. 2:**
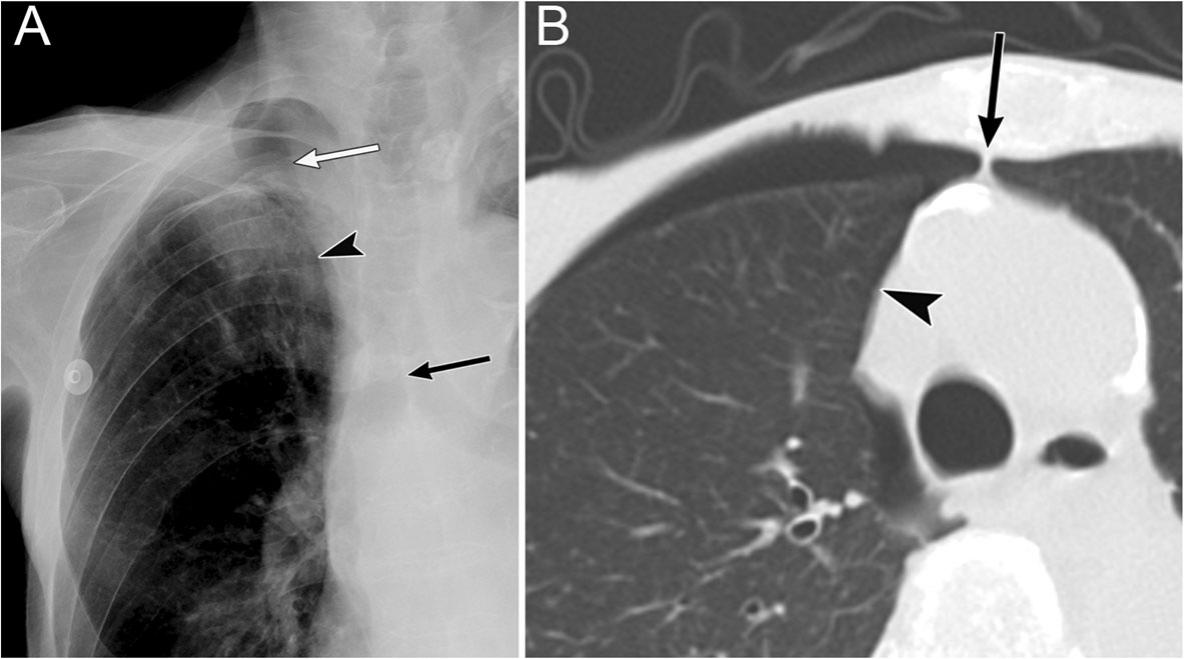
Pneumothorax in the suprahilar anteromedial space of an 83-year-old man with enteritis. **a** A supine radiograph shows sharp delineation of the superior vena cava (SVC) (arrowhead), right brachiocephalic vein (white arrow), and anterior junction line (black arrow). **b** Axial CT image at the level of the SVC shows air collection in the pleural cavity on the right sides of the SVC (arrowhead) and the anterior junction line (black arrow)

Radiographic signs of pneumothorax in the suprahilar anteromedial space include a sharp delineation of the superior vena cava (SVC) and the azygos vein on the right, sharp delineation of the left subclavian artery and the left superior intercostal vein on the left, and sharp delineation of the anterior junction line and the superior pulmonary vein on the affected side (Fig. 2) [19, 25]. The anterior junction line is a normal anatomical structure that is observed on chest radiography and is caused by the visceral and parietal pleurae of both lungs meeting anteriorly at the midline [26].

**Fig. 3:**
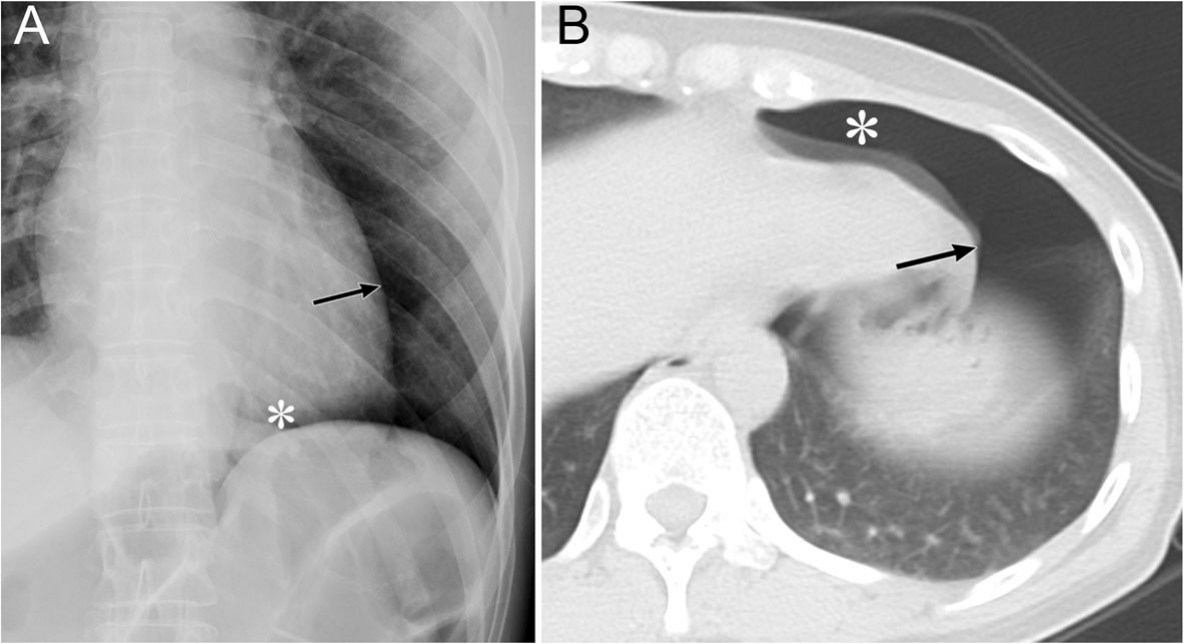
Pneumothorax in the infrahilar anteromedial space of a 39-year-old woman due to traffic accident-related trauma. **a** A supine radiograph shows a sharp delineation of the left heart border (arrow) and a deep anterior cardiophrenic sulcus (asterisk). **b** An axial CT of the lung base shows substantial air collection in the pleural cavity at the anterior cardiophrenic sulcus (asterisk) and on the left side of the heart border (arrow)

Radiographic signs of pneumothorax in the infrahilar anteromedial space include the sharp delineation of the heart border, inferior vena cava (IVC), pericardial fat pad, and deep anterior cardiophrenic sulcus (Fig. 3) [19]. The pericardial fat pad simulates a tumor or segmental lung collapse on radiography, which is easily differentiated with CT. The deep anterior cardiophrenic sulcus sign represents abnormal deepening and lucency of the anterior cardiophrenic sulcus due to air collection (Fig. 3). CT can clearly reveal air collection in the anterior cardiophrenic sulcus (Fig. 3). When the anteromedial space is large, the affected lung field may appear hyperlucent as compared with the opposite side and may also appear as a rounded or oval-shaped area of increased lucency (i.e., the “black oval”).

### Pneumothorax in the subpulmonary space (I-2)

The subpulmonary space is the second most common space of air collection in supine patients with pneumothorax [20]. Unfortunately, there is a lack of knowledge regarding the radiographic anatomy of this space, and this may result in the high failure rate associated with the detection of subpulmonary pneumothorax [19].

**Fig. 4:**
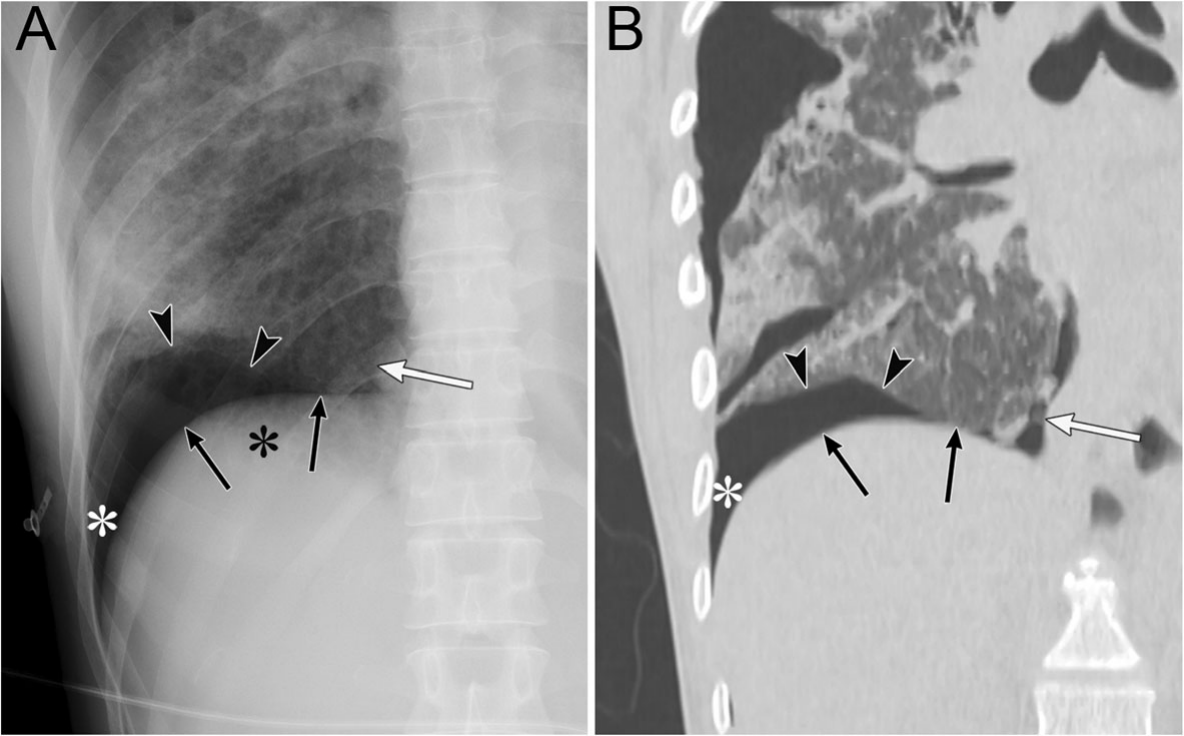
Pneumothorax in the subpulmonary space of a 36-year-old woman with interstitial pneumonia. **a** A supine radiograph shows a hyperlucent upper quadrant of the abdomen (black asterisk). The inferior surface of the lung (arrowheads), a sharply outlined diaphragm (black arrows), the sharp delineation of the inferior vena cava (IVC) (white arrow), and the “deep sulcus” sign (white asterisk) are also visible. **b** A coronal CT image clearly depicts the inferior surface of the lung (arrowheads)**,** a sharply outlined diaphragm (black arrows), the sharp delineation of the IVC, and the “deep sulcus” sign (white asterisk)

Radiographic signs of pneumothorax in the subpulmonary space include a hyperlucent upper quadrant of the abdomen, the visualization of the inferior surface of the lung, a sharply outlined diaphragm, the sharp delineation of the IVC on the right lung, the “deep sulcus” sign, and the “double diaphragm” sign (Fig. 4) [19, 27, 28]. In addition, the diaphragmatic dome becomes slightly elevated as the lung collapses.

The “deep sulcus” sign appears on radiographs as an abnormal deepening and lucency of the lateral costophrenic sulcus that extends toward the hypochondrium (Fig. 4) [19, 27, 28]. This sign is caused by air that collects in the lateral subpulmonary pleural space [19, 27, 28].

The “double diaphragm” sign is caused by the aerated lung that outlines the diaphragmatic dome and air in the pleural cavity that outlines the anterior costophrenic angle [27, 29]. Sagittal CT reveals air collection in the anterior costophrenic angle.

Pneumomediastinum and pneumoperitoneum may mimic subpulmonary pneumothorax. Unlike subpulmonary pneumothorax, the central portion of the diaphragm in these conditions may be outlined by air (i.e., the “continuous diaphragm” sign).

### Pneumothorax in the apicolateral space (I-3)

**Fig. 5:**
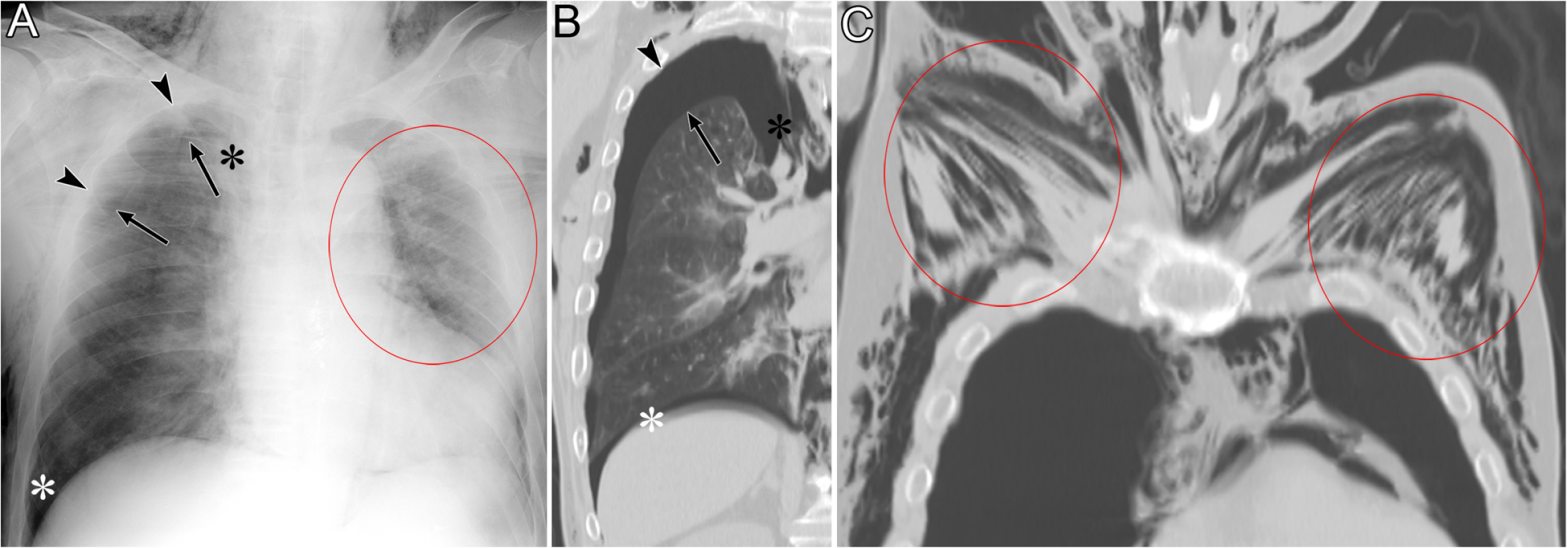
Pneumothorax in the apicolateral space of a 77-year-old man. Esophageal perforation caused by endoscopic submucosal dissection for esophageal cancer resulted in pneumomediastinum, pneumothorax, and subcutaneous emphysema. **a** A supine radiograph shows the visceral pleural line (black arrows) in the apicolateral space. The black arrowheads indicate the parietal pleura. Pneumothorax in the anteromedial space (black asterisk) and subpulmonary space (white asterisk), pneumomediastinum, and subcutaneous emphysema (red oval) are also visible. No vascular markings exist beyond the visceral pleural line. Subcutaneous emphysema in the pectoralis major muscle shows a linear disposition that follows the direction of the muscle fibers. **b** A coronal CT image reveals the visceral pleural line (black arrow) and the lack of any structures in the pleural cavity. The parietal pleura (arrowhead), pneumothorax in the anteromedial space (black asterisk), and pneumothorax in the subpulmonary space (white asterisk) are also observed. **c** A coronal CT image shows a linear disposition of subcutaneous emphysema that follows the direction of the muscle fibers of the pectoralis major muscle (red ovals)

The apicolateral space is a pleural recess where air anatomically does not easily accumulate in supine patients [20]. It is preceded by air collection in the anteromedial and subpulmonary space (Fig. 5) [19, 20].

A radiographic sign of pneumothorax in the apicolateral space is a thin visceral pleural line with no vascular markings beyond the line (Fig. 5) [19]. Vascular markings beyond the line are often observed when lung recoil differs in the presence of parenchymal diseases [19]. Visualization of the pleural line requires an aerated lung and air in the pleural cavity; therefore, the pleural line may not be visible in cases of pulmonary parenchymal diseases, such as pneumonia and acute respiratory distress syndrome (ARDS), pleural effusion, and focal pleural adhesions [19].

### Pneumothorax in the posteromedial space (I-4)

**Fig. 6:**
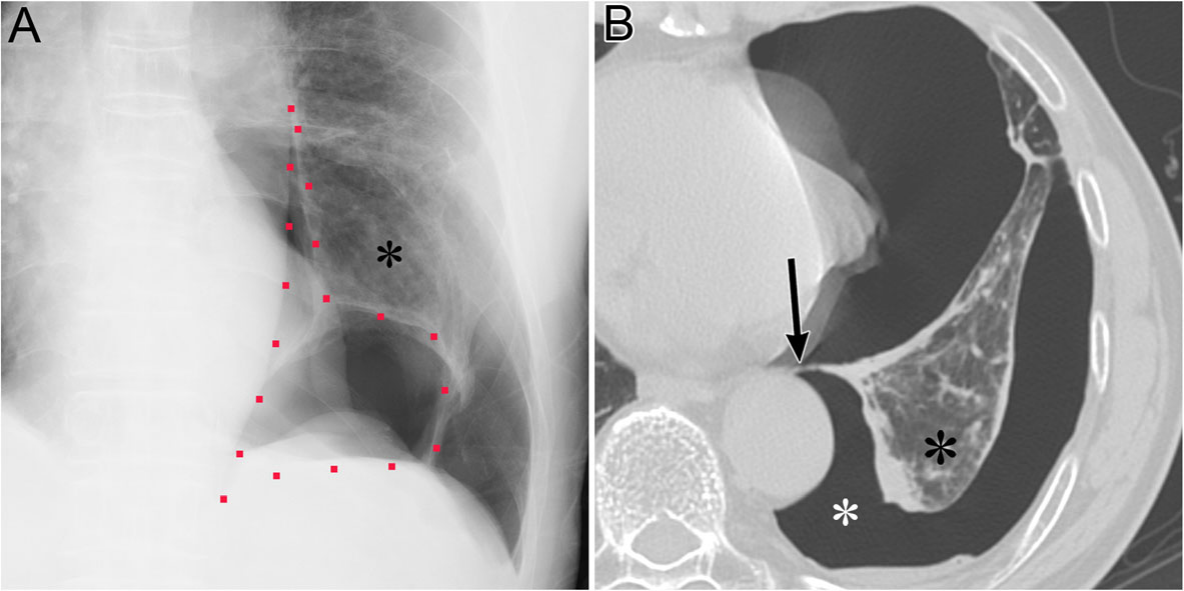
Pneumothorax in the posteromedial space of an 82-year-old man with chronic obstructive pulmonary disease. **a** A supine radiograph shows a lucent triangle (red dotted line) with a partially collapsed left lower lobe (black asterisk). The lucent triangle corresponds to the air collection space with its vertex in the hilum and a V-shaped base that delineates the costovertebral sulcus between the paraspinal line/descending aorta and the medial surface of the partially collapsed lower lobe. **b** An axial CT image of the lung bases shows pneumothorax in the posteromedial space (white asterisk). The left inferior pulmonary ligament (arrow), which is the linear structure between the mediastinum and the left lower lobe (black asterisk), are clearly visible

**Fig. 7:**
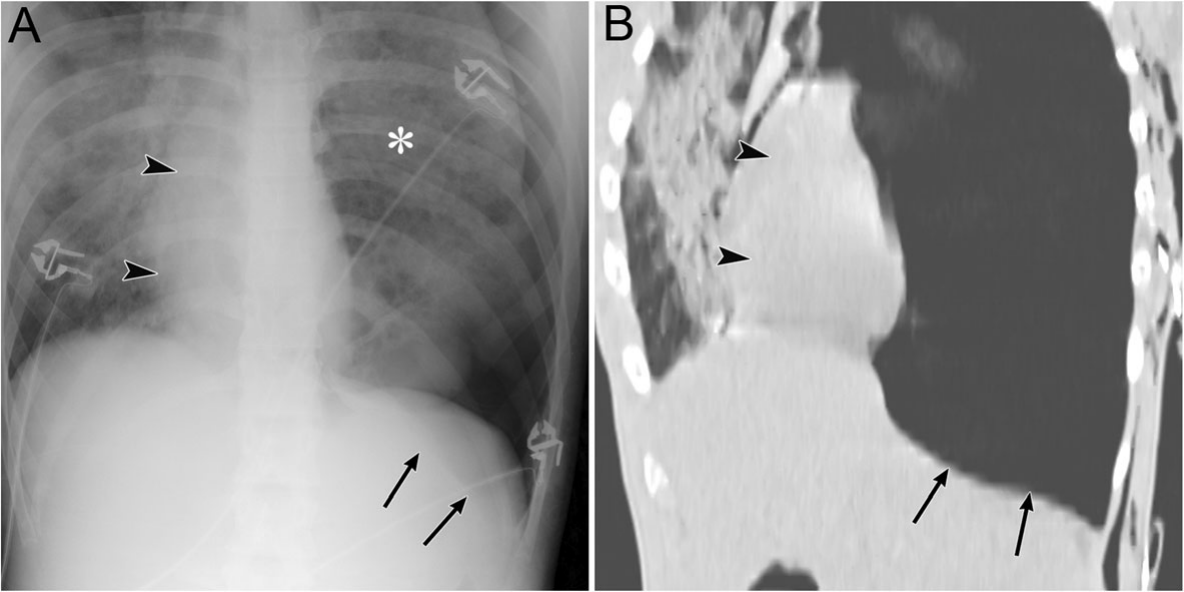
Tension pneumothorax due to traffic trauma in an 18-year-old man. **a** A supine radiograph shows left pneumothorax with a rightward mediastinal shift (black arrowheads) and partly inverted left hemidiaphragm (black arrows). The left lung is collapsed with a contusion (asterisk). **b** A coronal CT image clearly depicts a rightward mediastinal shift (black arrowheads) and an inverted left hemidiaphragm (black arrows)

Pneumothorax in the posteromedial space occurs in the presence of lower lobe collapse or parenchymal diseases [19, 20]. Radiographic findings include a lucent triangle with its vertex in the hilum and a V-shaped base that delineates the costovertebral sulcus (Fig. 6) [19, 20]. The medial surface of the triangle is the paraspinal line and descending aorta, and the lateral surface of the triangle is the medial surface of the collapsed lower lobe that is displaced from the midline [19, 20]. CT reveals a collapsed lower lobe that is anchored to the mediastinum by the inferior pulmonary ligament (IPL) and air collection in the posteromedial space (Fig. 7) [19]. Pneumomediastinum may mimic pneumothorax in the posteromedial space. The former often continues into the retroperitoneum or extends across the midline, whereas the latter remains above the diaphragm, ends at the costovertebral sulcus, and does not extend across the midline [19].

### Tension pneumothorax (I-5)

Tension pneumothorax is characterized by an abnormal increase in the pressure of the involved thoracic cavity. A one-way valve between the involved lung and the pleura leads to the continuous leakage of air into the pleural cavity and causes the accumulation of air within the pleural cavity [30]. Next, the ipsilateral lung collapses, the mediastinum is displaced away from the affected side, and the ipsilateral diaphragm is displaced downwards (especially in positive pressure ventilation) and may invert downwards [30]. The cardiac output is reduced because of the impaired venous return [30], and cardiac arrest eventually ensues [30]. Tension pneumothorax is a life-threatening event that occurs in the ICU; thus, it should be identified immediately, especially in patients who are treated with positive pressure ventilation. Immediate decompression of the thorax is mandatory in such cases [12].

Radiographic findings of the lung in tension pneumothorax are the same as those of the lung in a simple pneumothorax. In addition, mediastinal displacement, diaphragmatic inversion, increased intercostal space, and total or subtotal lung collapse reflect the expansion of the affected hemithorax (Fig. 7) [19, 31]. A more important sign of tension pneumothorax is the flattening of the heart border, SVC, and IVC [19]. This sign reflects impaired venous return [19].

### Mimics of pneumothorax (I-6)

The radiographic findings of many situations, such as skin folds, interlobar fissures, bullae in the apices, and air collection in intrathoracic extrapleural space [4, 12], mimic pneumothorax.

### Skin folds

When a portable chest radiography is conducted, the x-ray cassette is positioned behind the patient, and skin folds may occur between the chest wall and the cassette [4]. Skin folds are likely to occur in elderly and cachectic patients who have sagging skin.

**Fig. 8:**
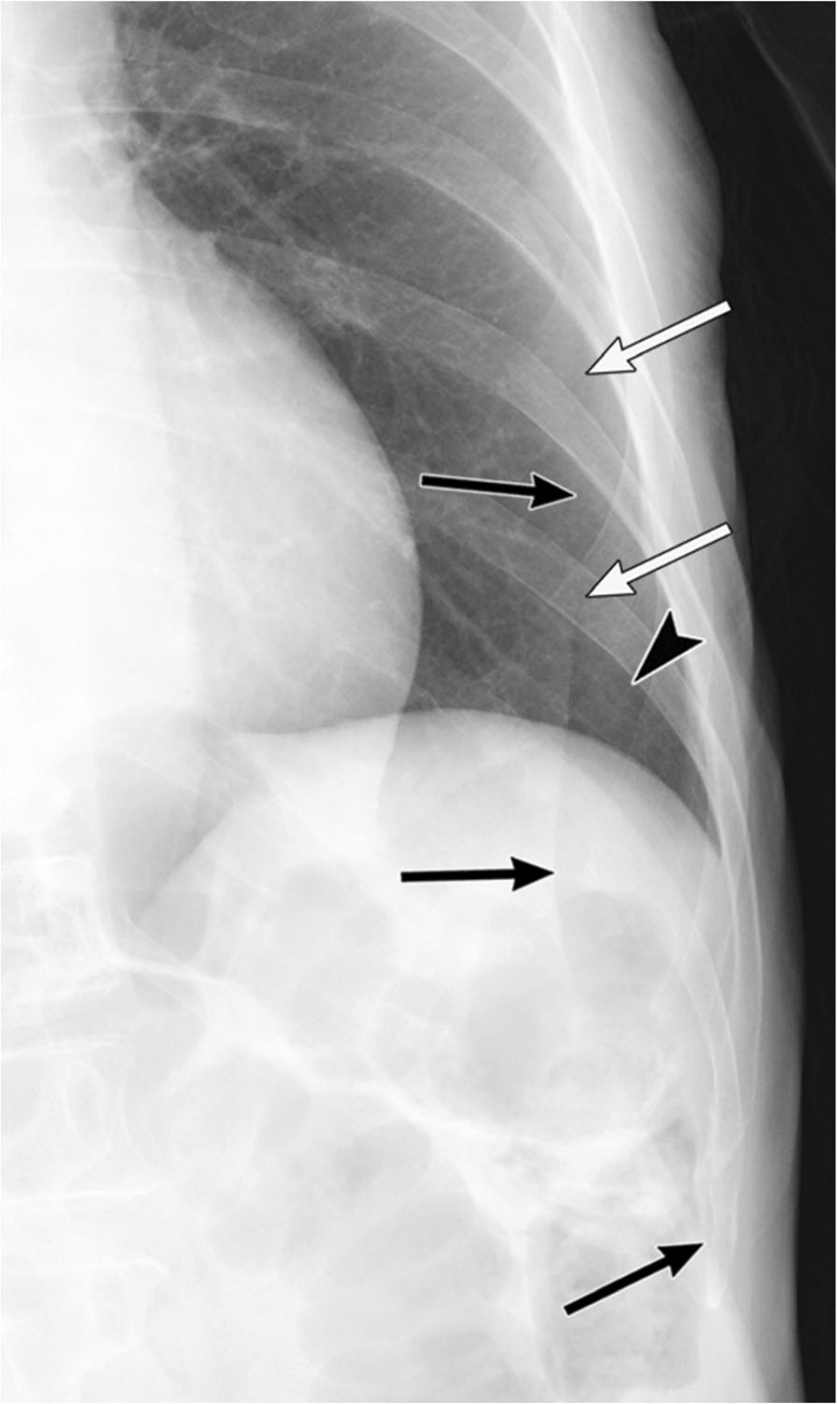
A skin fold mimicking pneumothorax on a radiograph in a 66-year-old woman with glioblastoma. The skin fold appears as a broad radiopaque band (black arrows) that is adjacent to a radiolucent band (white arrows), which reflects a negative Mach band effect in the left lung field on a supine radiograph. It extends beyond the pleural cavity. Vascular markings (black arrowhead) are visible beyond its edge

In contrast to the pleural line in pneumothorax, skin folds manifest as a broad radiopaque band that may extend beyond the parietal pleural line or midline with vascular markings beyond the lines of the folds (Fig. 8) (4, 12, 18, 31). Skin folds usually run in pairs or three at a time. Additionally, skin folds may be multiple or bilateral or may disappear suddenly [19]. A negative Mach effect enhancing the skin folds may be seen [19, 32]. Clothing or bed sheets may also produce a similar artifact [12].

### Interlobar fissures

**Fig. 9:**
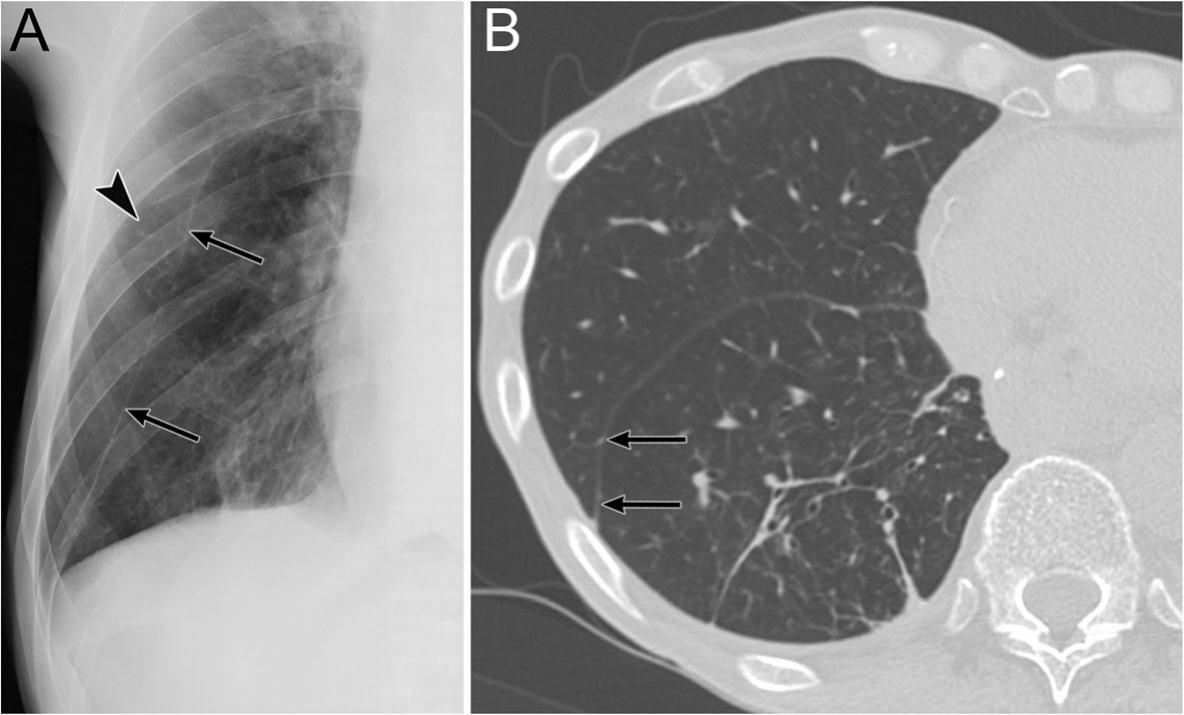
The major fissure mimicking pneumothorax on a radiograph in a 71-year-old man. He underwent partial resection of the right S6 for lung metastasis of esophageal cancer. **a** The major fissure appears as a thick linear opacity (black arrows) in the right lung field on a supine radiograph. It resembles the visceral pleura in pneumothorax. Unlike pneumothorax, vascular markings (black arrowhead) are visible outside of the thickened fissure. **b** An axial CT image shows the major fissure extending in the anteroposterior direction with a slight oblique angle (black arrows). Some volume loss in the right lower lobe caused the fissure to be displaced posteriorly; therefore, it seems to be visible on the anteroposterior radiograph

In the presence of interlobar pleural effusion or thickening of interlobar or accessory fissures, the interlobar fissures may resemble the visceral pleura seen in pneumothorax. Moreover, if a normal interlobar fissure is parallel to the x-ray beam, it may be visible and mimic the visceral pleura that is observed in pneumothorax on radiographs (Fig. 9). However, unlike pneumothorax, vascular markings are also visible outside of the fissure (Fig. 9).

### Bullae

**Fig. 10:**
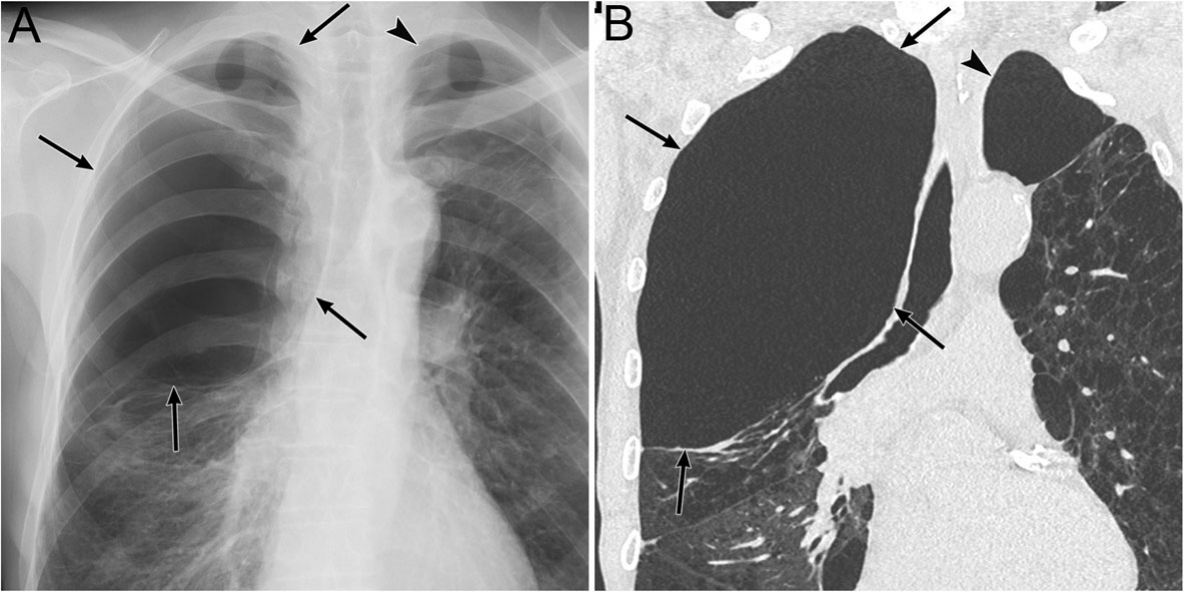
A giant bulla mimicking pneumothorax on a radiograph in a 67-year-old man. He has chronic obstructive pulmonary disease and a cigarette smoking habit. **a** A giant bulla appears as an oval radiolucency (black arrows) in the right lung apex on an erect radiograph. It resembles pneumothorax in the apicolateral space. Bullous emphysema exists in both lungs, and a large bulla is observed in the left lung apex (arrowhead). **b** A coronal CT image reveals a giant bulla (black arrows) in the right lung apex. A large bulla (black arrowhead) also exists in the left lung apex in addition to bullous changes in both lungs

Bullae, especially giant bullae, in the lung periphery can mimic pneumothorax (Fig. 10) [4]. A bulla is a well-defined airspace by a thin wall measuring more than 1 cm in diameter [21]. It is within the lung parenchyma and results from the destruction of the alveolar tissue. Additionally, bullae are often accompanied by emphysematous changes [21, 33]. Giant bullae occupy more than 30% of a hemithorax [34] and may be misdiagnosed as a pneumothorax even though the management of these two conditions differs [34]. Most patients with giant bullae with bullous emphysema are cigarette smokers, and usually present with progressive dyspnea over several months [33]. Conversely, patients with pneumothorax usually present with sudden dyspnea and chest pain.

Radiographic findings that are suggestive of bullae include the lack of a lung edge, round nature of the bullae convex to the lung, and presence of multiple bullae elsewhere [4]. Unlike the pleural line, the line demarcating a bulla is usually more horizontal [33].

## Abnormal air collection in the chest wall

Subcutaneous emphysema and air collection in the intrathoracic extrapleural space are diseases that are associated with air collection in the chest wall. Since the intrathoracic extrapleural space and the pleural cavity are anatomically close, air collection in the intrathoracic extrapleural space resembles pneumothorax on radiographs.

### Subcutaneous emphysema (II-1)

Subcutaneous emphysema occurs when air accumulates at the level of subcutaneous fatty tissue and superficially to the deep fascia that covers the skeletal muscle planes (Fig. 1) [5]. The most common clinical symptom of subcutaneous emphysema is swelling around the neck accompanied by pain in the chest [35]. Crepitus can be typically felt by physical examinations. Trauma and iatrogenic complication (after a surgical procedure or insertion of a chest tube, etc.) are its common causes. Infectious diseases can also cause it [5]. Abnormal air comes from outside or inside of the body (pneumothorax, pneumomediastinum, PIE, retroperitoneal gas, etc.) or from gas-forming microbes (necrotizing fasciitis, Fournier gangrene, etc.) [5, 35]. Subcutaneous emphysema often causes minimal symptoms, is not critical, and does not require a specific treatment [35]. Accordingly, treatment is targeted to the underlying cause of the condition.

Subcutaneous emphysema appears as multiple lucencies in the subcutaneous tissue on radiographs. When air involves deeper tissue muscles, a characteristic radiographic finding is observed as linear lucencies with a linear disposition that follow the direction of the fascial planes and/or muscle fibers (Fig. 5) [5]. Multiple lucencies in the soft tissue can mimic alveolar infiltration. By contrast, true pulmonary parenchymal diseases or pneumothorax may be obscured by subcutaneous emphysema. Superposition of external structures (e.g., long hair) showing linear radiolucency may resemble subcutaneous emphysema [5].

### Air collection in the intrathoracic extrapleural space (II-2)

The extrapleural space is anatomically part of the chest wall and lies between the parietal pleura and the inner surface of the ribs. It contains extrapleural fat, endothoracic fascia, and the innermost intercostal muscle (Fig. 1) [13, 36]). The normal extrapleural space is not distinguishable as separate structures on CT images [13, 36], and it can extend into the mediastinum [13]. Air collection in the extrapleural space is caused by barotrauma, disruption of the tracheobronchial and esophagus, and the extension of air from other space, such as the neck and retroperitoneum [13]. Although this condition is usually managed with close observation, other treatments may be needed in cases of tracheobronchial tree or esophageal lesions [13].

**Fig. 11:**
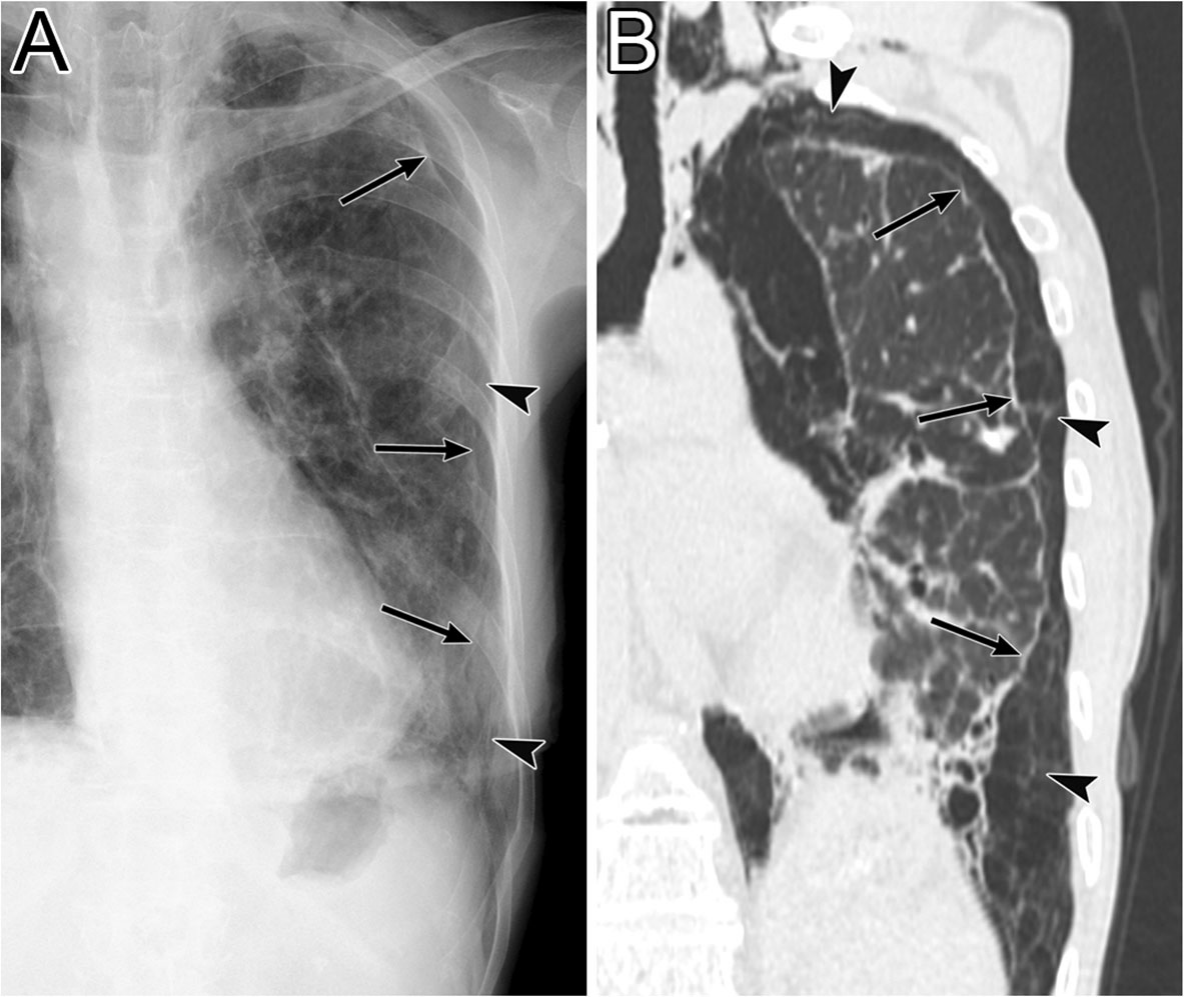
Air collection in the intrathoracic extrapleural space of a 64-year-old woman. She has Sjögren’s syndrome and interstitial pneumonia. **a** A supine radiograph shows a linear opacity (black arrows) in the left peripheral lung field that resembles pneumothorax. Fascial webs (black arrowheads) are visible outside of the linear opacity; this finding proves that air does not exist in the pleural cavity but collects in the intrathoracic extrapleural space. The linear opacity is composed of both the visceral and parietal pleura. Pneumomediastinum and subcutaneous emphysema are also visible. **b** A coronal CT image clearly depicts fascial webs (black arrowheads) in the intrathoracic extrapleural space. The mixture of the visceral and parietal pleura (black arrows) is slightly wavier than the typical visceral line in pneumothorax

Air collection in the extrapleural space appears on radiographs as a lucency, such as visible fascial webs bounded by the pleural line (i.e., consisting of the parietal and visceral pleura) (Fig. 11) [6, 13]. Air collection in the extrapleural space resembles pneumothorax, and the differential diagnosis is very important to avoid unnecessary thoracentesis (Fig. 11) [6, 13]. Some radiographic findings can help their differentiation. With air collection in the extrapleural space, visible fascial webs are detected outside of the pleural line and air distribution does not change with patients’ positions (Fig. 11) [6, 13]. The pleural line is wavier in air collections in the extrapleural space than the typical visceral line in pneumothorax. CT allows for the clear visualization of fascial webs outside of the pleural line.

## Abnormal air collection in the mediastinum

**Fig. 12:**
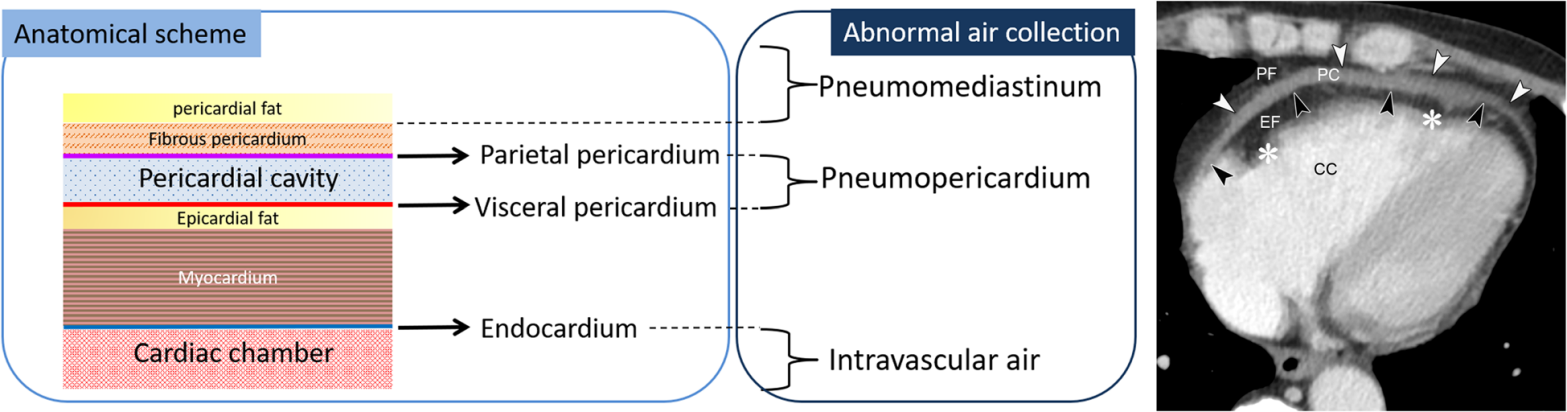
Anatomy of the pericardium and related diseases of abnormal air collection at each anatomical space. Axial chest CT of a 47-year-old man with chronic kidney disease shows fluid collection in the pericardial cavity. The CT shows the pericardial fat (PF), parietal pericardium (white arrowheads), pericardial cavity (PC), visceral pericardium (i.e., epicardium) (black arrowheads), epicardial fat (EF), myocardium (white asterisks), and cardiac chamber (CC)

Pneumomediastinum and IPL air collection are diseases in which air collects in the mediastinum. Figure 12 shows the CT anatomy of the mediastinum and the pericardium and diseases of abnormal air collection in each anatomical space. The mediastinum and the pericardium are anatomically close; thus, pneumomediastinum resembles pneumopericardium on radiographs. Intravascular air is the abnormal collection of air in the cardiac chamber and great vessels, and it is mainly caused by iatrogenic complications, including intravenous injections, central venous catheters, lung biopsies, and marker placements. Although intravascular air can be detected by CT, it may be difficult to identify on radiographs unless it is present in large amounts.

### Pneumomediastinum (III-1)

Pneumomediastinum represents air in the mediastinum except for the lumens of the esophagus and airway [6–8]. Pneumomediastinum has many causes. Air comes from the alveoli, tracheobronchial trees, and esophagus, or from the passage of abnormal extraluminal gas into the thorax from the neck, retroperitoneum, or chest wall [6–8]. Pneumomediastinum can cause pneumothorax, pneumopericardium, pneumoperitoneum, and pneumoretroperitoneum [7, 8]. The treatment of pneumomediastinum differs depending on its cause; therefore, it is necessary to detect the exact cause using CT (especially in cases of tracheal/esophageal rupture).

**Fig. 13:**
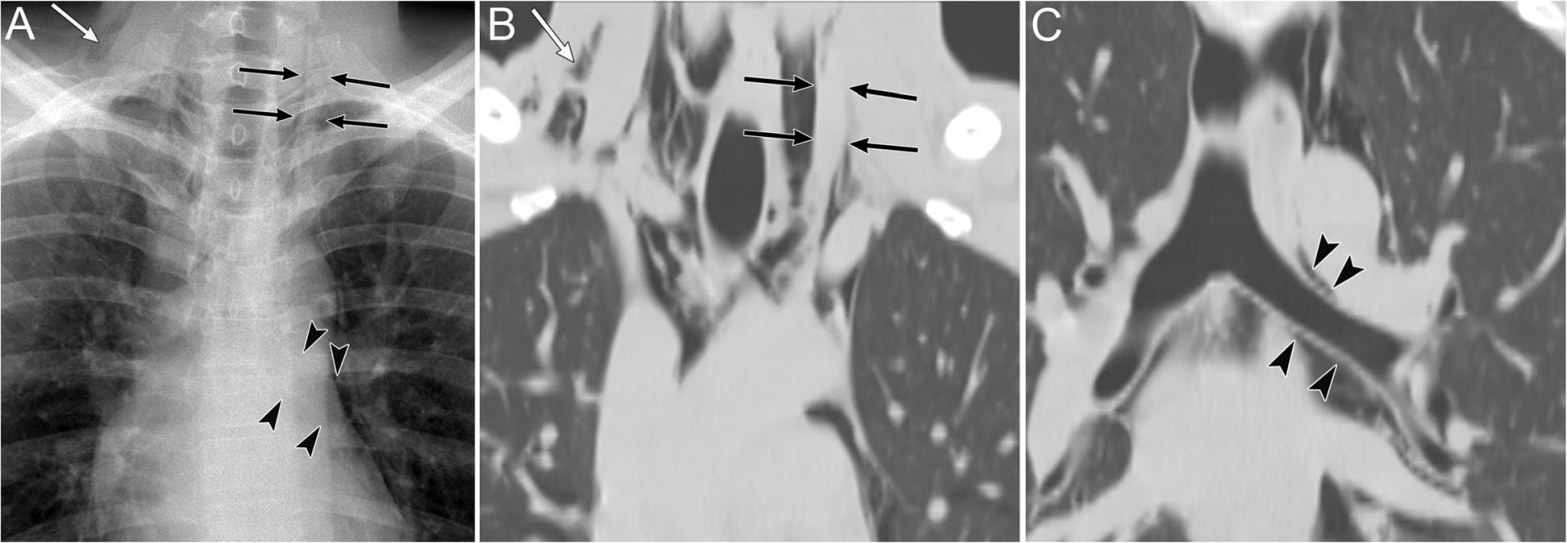
Pneumomediastinum due to chronic cough and vomiting in a 22-year-old man. **a** A supine radiograph shows the “tubular artery” sign (black arrows) and the “double bronchial wall” sign (black arrowheads). Subcutaneous emphysema is also evident (white arrow). **b** A coronal cervical CT image shows the “tubular artery” sign (black arrows). Both sides of the left internal jugular vein are visible due to pneumomediastinum. Subcutaneous emphysema is also visible (white arrow). **c** The coronal CT image at the carina level shows the “double bronchial wall” sign (black arrowheads). Both sides of the left bronchial wall are visible because of air in the bronchus and pneumomediastinum surrounding the wall

**Fig. 14:**
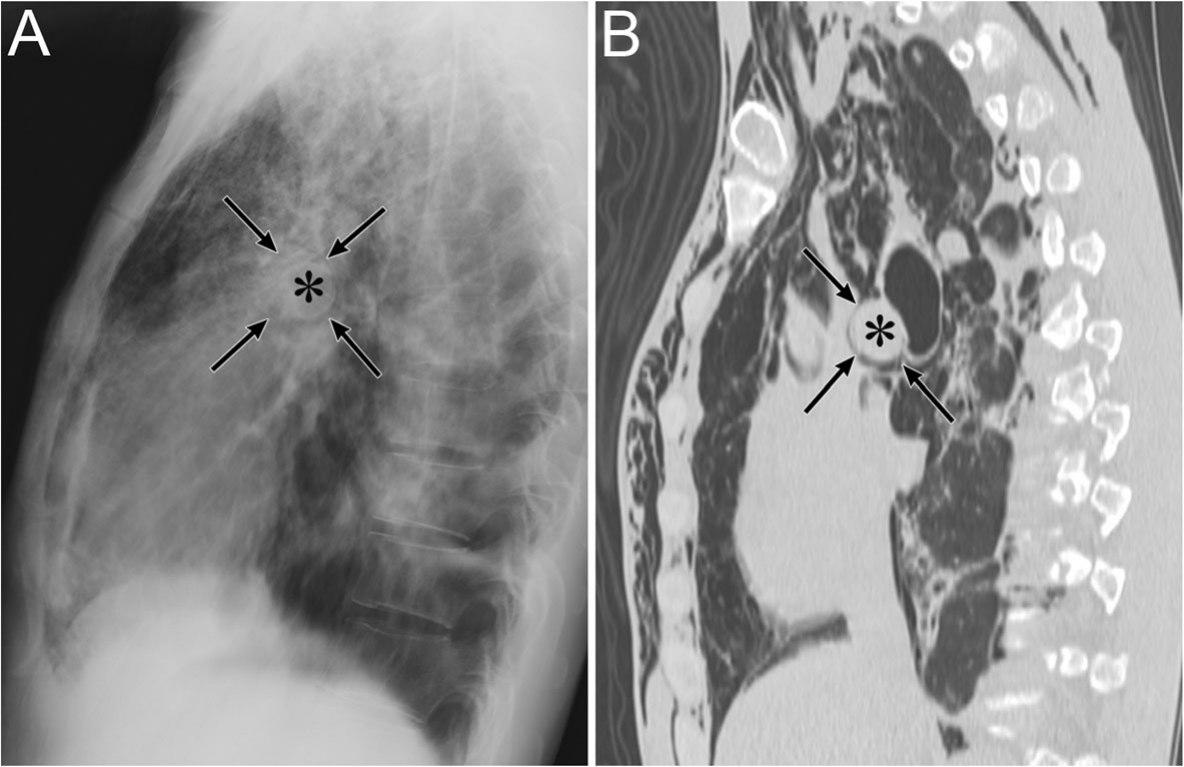
Pneumomediastinum of a 43-year-old man with acute myelogenous leukemia. **a** Erect lateral radiograph shows the “ring around the artery” sign. **b** A sagittal CT image also shows a lucent ring (arrows) surrounding the right pulmonary artery (asterisk), a feature called the “ring around the artery” sign

**Fig. 15:**
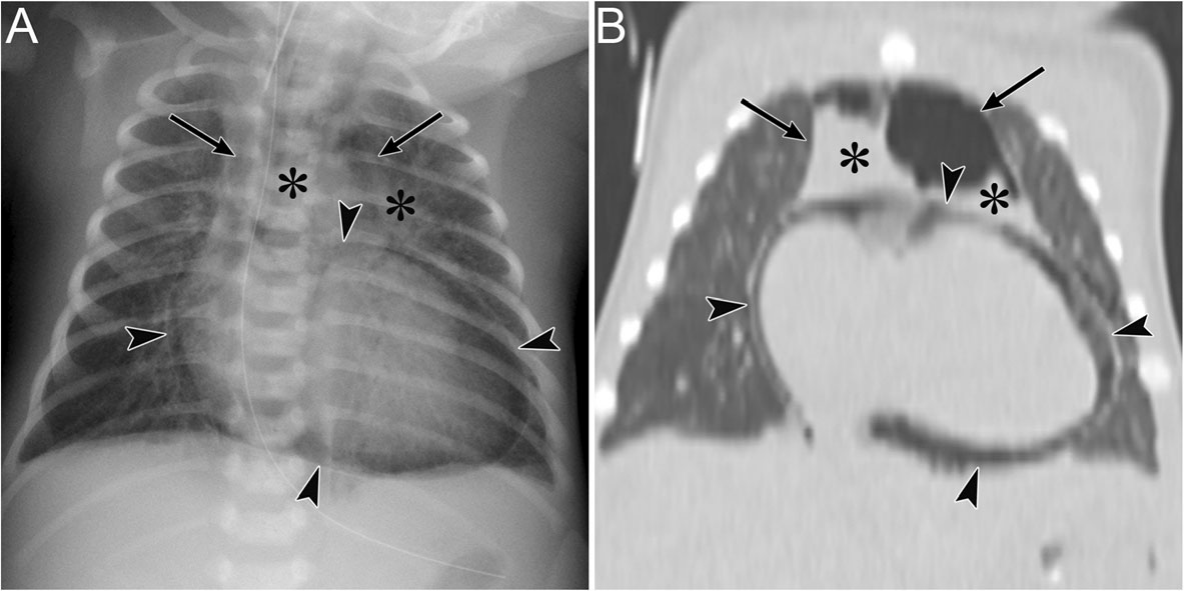
Pneumomediastinum and pneumopericardium in a 3-day-old girl with pulmonary atresia. Pneumomediastinum is caused by the obstruction and check valve effect of the tracheal tube with pulmonary secretions. **a** A supine radiograph shows the “spinnaker sail” sign with thymus elevation (asterisks) by pneumomediastinum (arrows). Pneumopericardium (arrowheads) is also visible. **b** A coronal CT image reveals pneumomediastinum (arrows) surrounding the thymus (asterisks) and pneumopericardium (arrowheads)

**Fig. 16:**
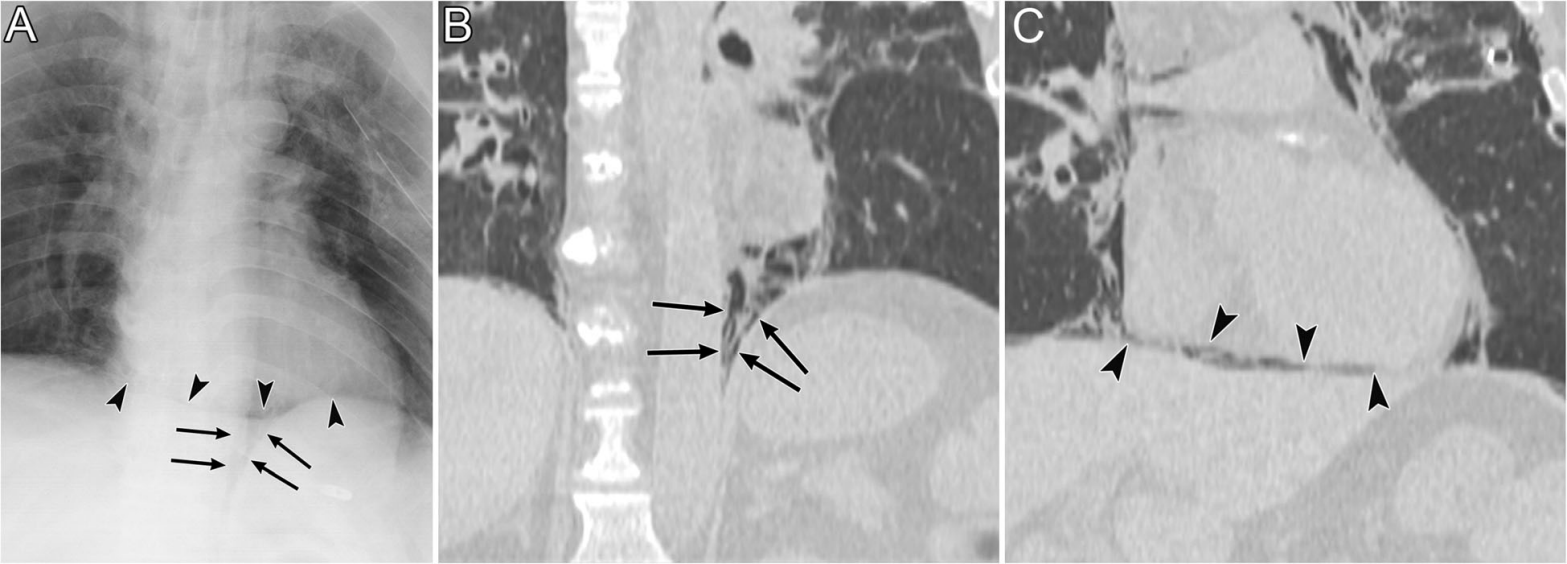
Pneumomediastinum in a 50-year-old man. The pneumomediastinum was caused by tracheostomy displacement. **a** A supine radiograph shows pneumomediastinum and subcutaneous emphysema. Air along the left aortic border and the medial left hemidiaphragm become V-shaped (i.e., “Naclerio’s V” sign) (arrows). The “continuous diaphragm” sign (arrowheads) is also visible. **b** A coronal CT image of the lung base reveals “Naclerio’s V” sign (arrows). **c** Another coronal CT image of the lung base reveals a “continuous diaphragm” sign (arrowheads)

Pneumomediastinum appears as radiolucent lines or bubbles outlining the mediastinal structures that are not normally visible or as an outwardly elevated mediastinal pleura [6–8]. In particular, the mediastinal air may outline the medial border of the SVC, right innominate artery, left common carotid artery, and left subclavian artery [8]. The radiographic signs of pneumomediastinum include the “tubular artery” sign, “ring around the artery” sign, “double bronchial wall” sign, “spinnaker sail” sign, “continuous diaphragm” sign, and “Naclerio’s V” sign [6–8, 37]. The “tubular artery” sign is the visualization of both sides of a vessel in a pneumomediastinum or aerated lung (Fig. 13) [8]. “A ring around the artery” sign is seen on lateral chest radiographs and appears as a lucent ring that surrounds the right pulmonary artery (Fig. 14) [6–8, 37]. The “double bronchial wall” sign is the visualization of both sides of the bronchial wall due to air in the bronchus and air surrounding the wall (Fig. 13) [8]. The “spinnaker sail” sign is described as thymus elevation by pneumomediastinum (Fig. 15) [7] and is usually found in children [7]. The “continuous diaphragm” sign is a classic sign of pneumomediastinum that is observed on frontal radiographs when gas in the mediastinum separates the pericardium and superior surface of the diaphragm across the midline (Fig. 16) [6–8]. “Naclerio’s V” sign was first reported by Naclerio [38] as a radiographic sign of spontaneous esophageal rupture. This sign is described as V-shaped air collection that consists of air along the left aortic border and medial left hemidiaphragm (Fig. 16) [6, 7]. These signs can be clearly detected on CT.

Pneumothorax in the anteromedial space or pneumopericardium may exhibit similar to the radiographic findings of patients with pneumomediastinum. Unlike pneumothorax, pneumomediastinum can spread to the soft tissue of the neck and face and to the retroperitoneal space, outlines the mediastinal structures, and will not change in distribution when the patient’s position changes [6]. Intra-aortic balloon pumping may also resemble pneumomediastinum. An oval lucency along the aorta reflecting air within the balloon accompanied by a catheter caudally is a characteristic imaging finding.

Tension pneumomediastinum is a rare life-threatening condition [14, 39] that is associated with mechanical ventilation. Increased intramediastinal pressure compresses the great vessels and reduces venous return, stroke volume, and cardiac output [14, 39]. In cases of cardiorespiratory embarrassment, mediastinal decompression (e.g., percutaneous mediastinal drainage) is necessary for treatment [14].

Although it is not difficult to detect pneumomediastinum on radiographs, it is difficult to diagnose tension pneumomediastinum on radiographs. The “Earth-Heart” sign was recently reported to be useful for the diagnosis of tension pneumomediastinum [39]. In this sign, the cardiac silhouette resembles the shape of an oblate sphere, such as the Earth, on a radiograph [39]. Tension pneumomediastinum causes cardiac compression; therefore, the transverse cardiac diameter increases and the vertical cardiac diameter decreases [39].

### Air collection in the IPL (III-2)

**Fig. 17:**
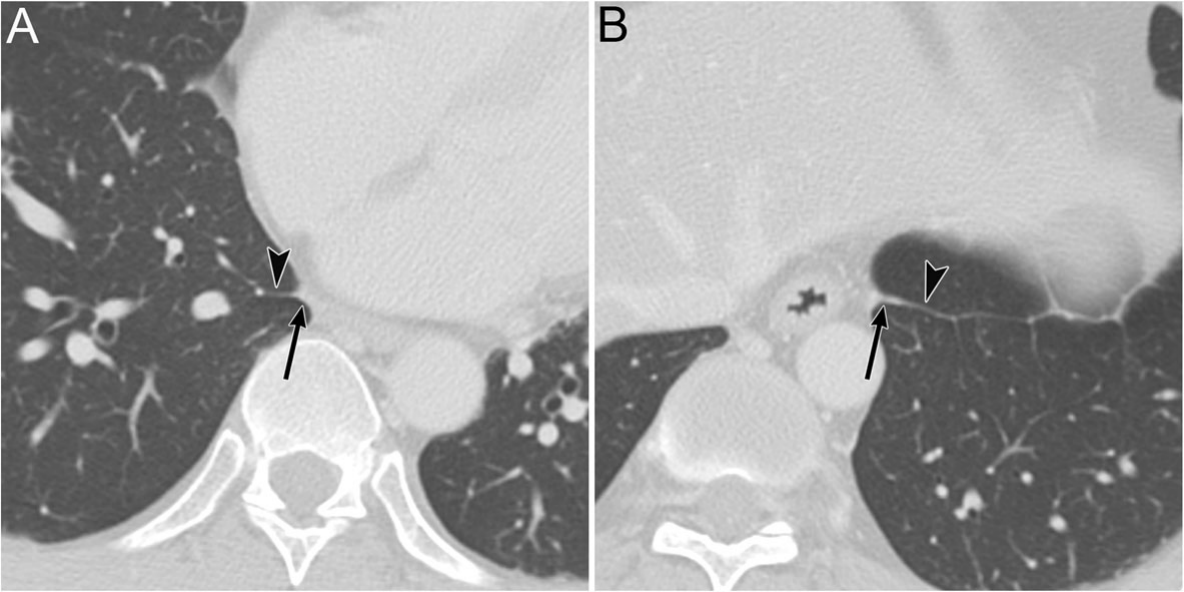
The normal inferior pulmonary ligament (IPL) on CT. A 48-year-old man with chronic obstructive pulmonary disease. **a** An axial CT image of the right lung at the lung base shows the right IPL (black arrow). It appears as a beak that extends laterally from the mediastinum (between the IVC and the azygos vein) with the intersublobar septum (black arrowhead)**. b** An axial CT image of the left lung at the lung base shows the left IPL (black arrow). It appears as a beak that extends laterally from the mediastinum (along the esophagus) with the intersublobar septum (black arrowhead)

The IPL is a triangular sheet of parietal and visceral pleura that extends from the hilum to the diaphragm and from the mediastinum to the medial surface of the lower lobe. The connective tissue of the IPL is continuous laterally with the interstitial tissue of the lung [40, 41]. On CT, the normal IPL appears as a beak that extends laterally from the mediastinum, and the intersublobar septum extends to the pulmonary vein (Fig. 17) [40]. The right IPL lies between the IVC and the azygos vein, and the left IPL lies along the esophagus (Fig. 17) [40].

The IPL air collections come from pneumomediastinum secondary to the rupture of the distal esophagus [8] or from PIE that may occur with alveolar rupture caused by barotrauma [41]. As the pneumomediastinum resolves, IPL air collection also decreases [42].

**Fig. 18:**
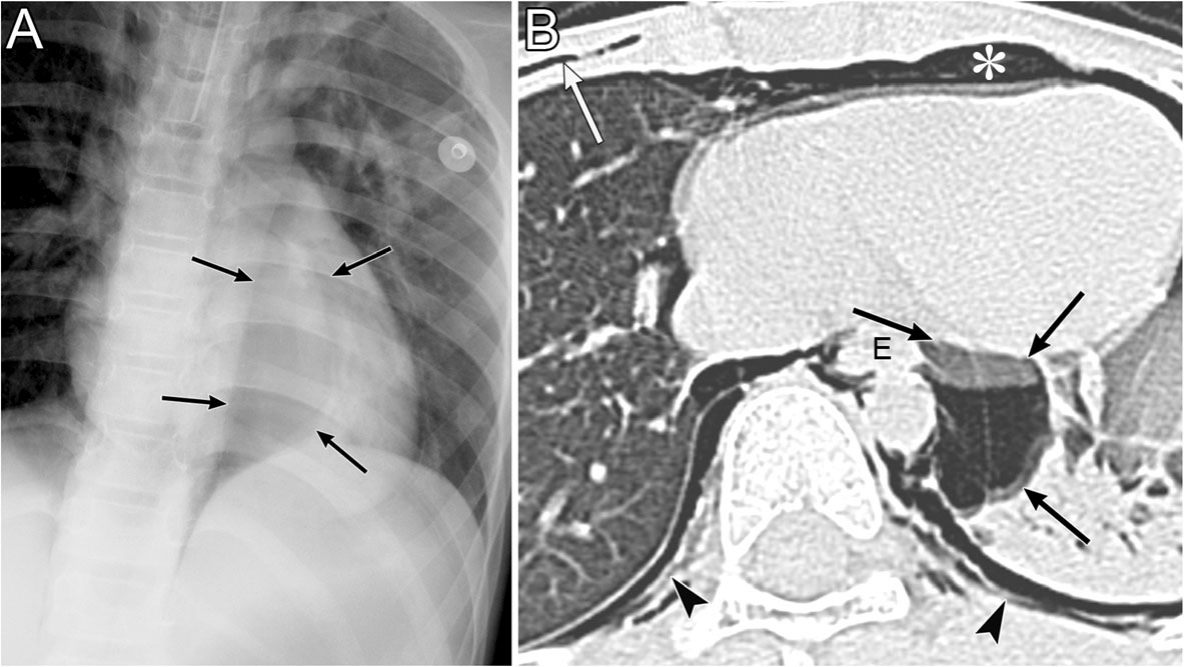
Air collection in the inferior pulmonary ligament (IPL) of an 8-year-old boy with traumatic tracheal injury. **a** A supine radiograph shows an oval lucency (black arrows) in the left paramediastinal area, which reflects air collection in the left IPL. **b** An axial CT image reveals air collection in left IPL (black arrows) because air collects along the mediastinum at the level of the esophagus (E). Pneumomediastinum (white asterisk), intrathoracic extrapleural space air collection (black arrowheads), and subcutaneous emphysema (white arrow) are also evident

**Fig. 19:**
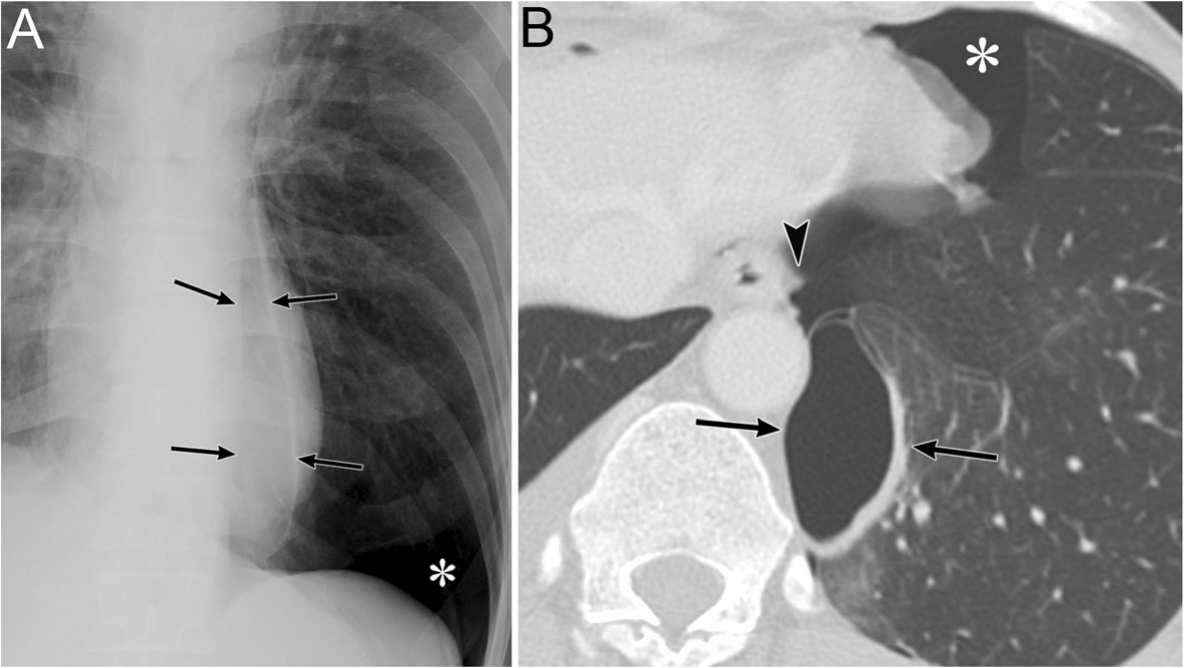
A pneumatocele mimicking inferior pulmonary ligament (IPL) air collection of a 61-year-old man. He has right main bronchial stricture treated under positive pressure ventilation. **a** A supine radiograph shows an oval lucency (black arrows), which represents a left lower lobe pneumatocele, in the left paramediastinal area. A pneumothorax (white asterisk) is also visible, but the pneumomediastinum is not present. **b** An axial CT image reveals a left lower lobe pneumatocele (black arrows), while the normal left IPL (black arrowhead) lies along the esophagus. A pneumothorax (white asterisk) is also visible. The pneumatocele and pneumothorax disappeared on the supine radiograph 3 months later

IPL air collection appears as an oval lucency in the paramediastinal area on a frontal radiograph (Fig. 18) [41] and as an oval lucency behind the cardiac silhouette on a lateral radiograph [41]. Pneumatocele in the paramediastinal area and pneumothorax in the posteromedial space radiographically mimic IPL air collection (Fig. 19 )[43]. In contrast to IPL air collection, a pneumatocele and pneumothorax may extend over the hilum or may be posterior to the IPL [43]. IPL air collection is usually accompanied by pneumomediastinum or PIE (Fig. 19) [41], and CT is useful for distinguishing between these diseases. In cases of a pneumatocele or pneumothorax, a normal IPL may be identified by CT (Fig. 19).

## Abnormal air collection in the pericardium [pneumopericardium (IV-1)]

Pneumopericardium is a rare but potentially life-threatening condition in which air enters the pericardial cavity. The visceral pericardium is the membranous layer that covers the myocardium and epicardial fat. It also reflects back near the origin of the major vessels and is continuous with the parietal pericardium (Fig. 12). The pericardial cavity is the space between both pericardia (Fig. 12), and each pericardium is observed as a thin linear structure on CT. Each pericardium is often not visualized over much of the left ventricle where a small amount of pericardial and epicardial fat exists and because motion artifacts of the left ventricle degrade image quality [44]. The pericardial cavity normally contains a small amount of fluid (between 15 and 50 mL) [44]. Pneumopericardium more commonly occurs in the pediatric population [45]. The main clinical symptom of pneumopericardium is chest pain, and radiating pain, dyspnea, and palpitations may also occur [9, 45]. Pneumopericardium may be suspected based on a pericardial “crunch” on auscultation [46] and is caused by trauma, iatrogenic complications (e.g., thoracotomy, thoracentesis, etc.), fistula to the pericardium with the bronchus or esophagus, barotrauma, pericardial infection, and congenital defect of the pericardium [6, 9, 47]. Pneumopericardium may impair right ventricular filling and subsequently cause pericardial tamponade with hypotension and cardiogenic shock. Rapid fluid resuscitation and emergent pericardiocentesis should be administered when cardiac tamponade occurs [9]. The diagnosis of pneumopericardium can be confirmed by radiography, CT, and echocardiography [9]. Pneumopericardium shows air microbubbles in the pericardial cavity on echocardiograms [48].

Pneumopericardium appears as a continuous, broad-band, radiolucent stripe along the cardiac border with a fine line that represents the pericardial sac (Fig. 15) [6, 9]. Its radiographic findings may resemble those of pneumomediastinum [6, 9]. Differential diagnosis of these entities is important because their treatment differs. Some radiographic features help distinguish pneumopericardium from pneumomediastinum. Pneumopericardium appears as a single broad band [6, 9], whereas pneumomediastinum appears as multiple thin streaks [6, 9]. Owing to the anatomical limits of the pericardium, pneumopericardium does not extend to the aortic arch or neck along the tracheobronchial tree [6, 9]. Additionally, unlike pneumomediastinum, the distribution of pneumopericardium changes according to patient’s positions [6, 9].

## Abnormal air collection in the lung

**Fig. 20:**
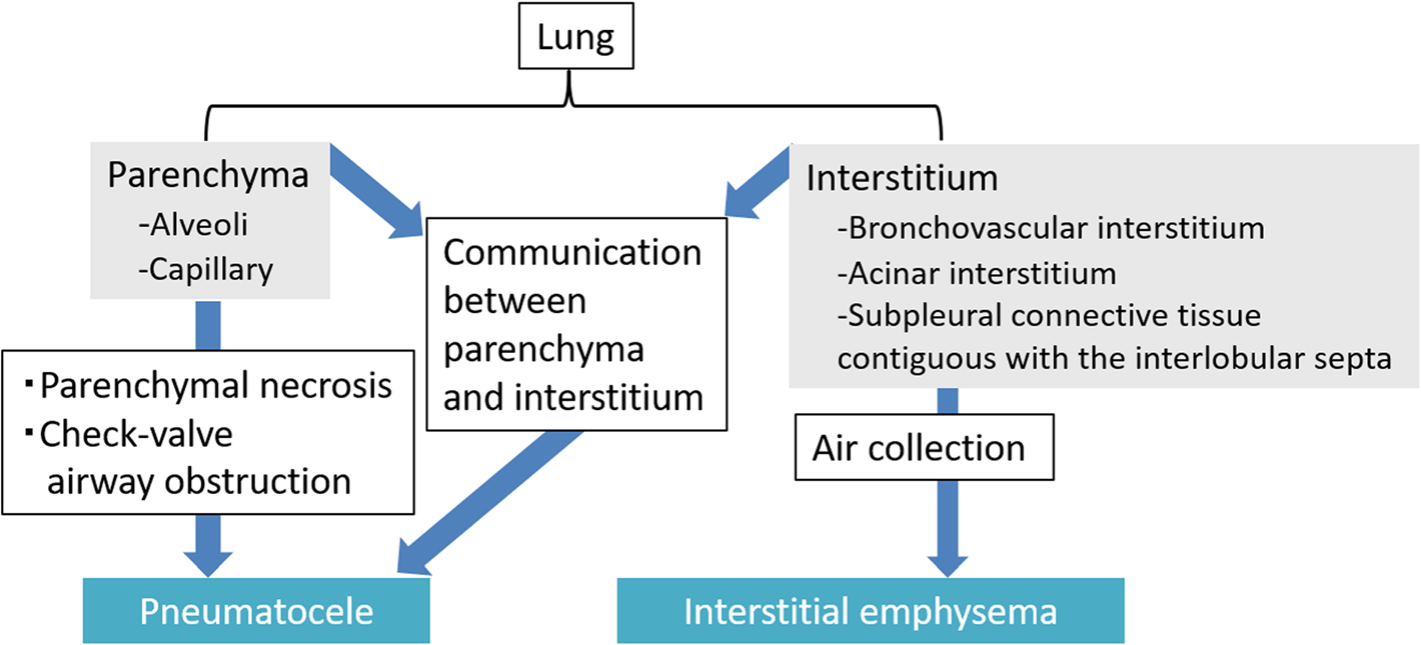
Anatomy of the lung and diseases of abnormal air collection in each anatomical space

PIE and pneumatocele are diseases in which air collects in the lung. Figure 20 shows the anatomy of the lung and diseases of abnormal air collection in each anatomical space. The lung is anatomically composed of the parenchyma (i.e., the alveoli and capillaries) and interstitium [21] (Fig. 20). The pulmonary interstitium is composed of the bronchovascular interstitium, the acinar interstitium, and the subpleural connective tissue that is contiguous with the interlobular septa [21] (Fig. 20).

### PIE (V-1)

PIE is a potentially life-threatening condition wherein air collects in the pulmonary interstitium [16]. Although PIE commonly occurs in neonates but rarely in adults [16, 49]. Greenough et al. [50] reported that the incidence and mortality rates of PIE in preterm infants who required ventilation for respiratory distress syndrome (RDS) were 19.5% and 24%, respectively [50]. PIE is caused by air leakage due to alveolar rupture in barotrauma [49, 51], and it is associated with intermittent positive pressure ventilation with peak airway pressures exceeding 30 cm H_2_O, severe ARDS, and pulmonary abnormalities, including pneumonia, coughing, chest trauma, and asthma [16, 49, 51]. The continued air leakage from the alveoli in patients with PIE leads to subpleural cysts, pneumomediastinum, IPL air collection, pneumothorax, and subcutaneous emphysema [49]. When PIE becomes extensive, it may compress the adjacent lung, heart, great veins, and pulmonary vessels. The pulmonary vascular resistance will increase and cardiac output may decrease [26]. When accompanied by pneumothorax, the mortality rate of PIE doubles in infants with RDS [43]. The main treatment is to achieve adequate oxygenation with a lower mean and peak airway pressure of ventilation [20]. Lateral decubitus positioning with the affected lung in the dependent position is also a useful treatment for localized disease [16, 52], and selective main bronchial intubation of the unaffected side is useful for the unilateral disease [16, 53].

**Fig. 21:**
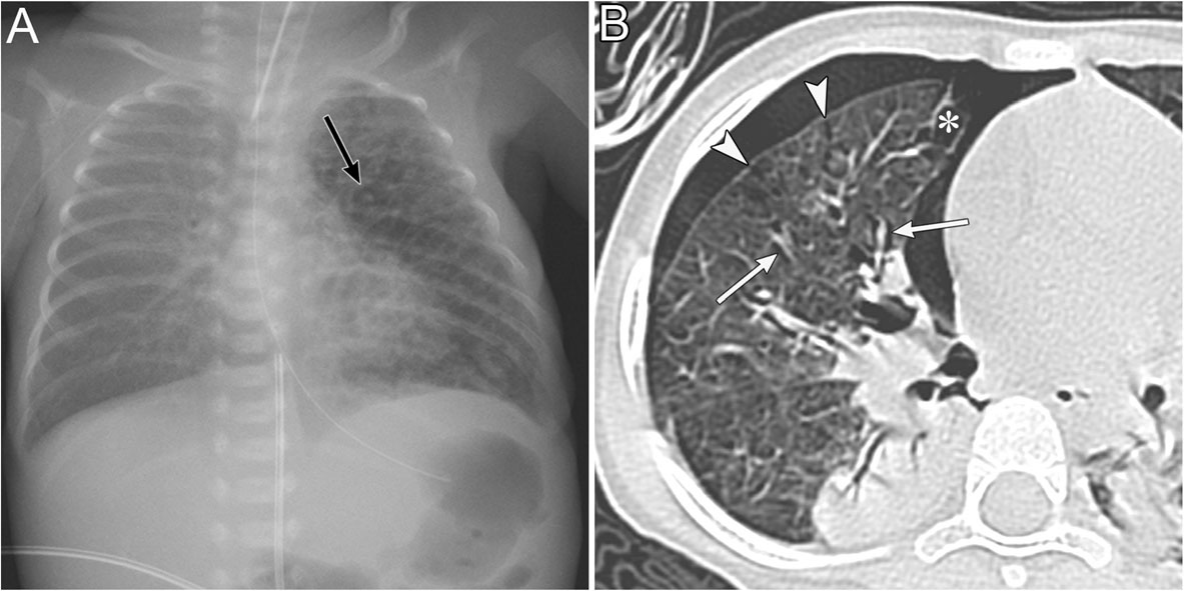
Pulmonary interstitial emphysema. **a** A supine radiograph of a 6-day-old girl with an extremely low birth weight shows generalized irregular radiolucent mottling and perivascular air (black arrow) in the left lung. **b** An axial chest CT image of a different patient (a 20-month-old girl with pneumonia) shows a subpleural cyst (asterisk), air within the interlobular septa (arrowheads), perivascular and peribronchial emphysema (white arrows), and pneumothorax

PIE appears radiographically as lucent streaks that radiate from the hila to the periphery of the lung. This finding reflects peribronchovascular air and air within the interlobular septa that resembles an air bronchogram (Fig. 21) [16, 49, 51]. The lucent streaks of PIE tend to be more numerous and more irregular on a radiograph than those of an air bronchogram [49]. Irregular radiolucent mottling in the lung reflects subpleural cysts and parenchymal cysts (Fig. 21) [16, 49, 51]. On a CT image, PIE is visualized as perivascular and peribronchial emphysema, air within the interlobular septa, and subpleural cysts (Fig. 21) [16, 49, 51]. Rapid changes in the distribution and extent of air are characteristic of PIE [36], and it may disappear within 1 to 2 days [49].

### Pneumatocele (V-2)

A pneumatocele is a thin-walled, air-filled space in the lungs [21]. The causes of pneumatocele include severe pneumonia, thoracic trauma, hydrocarbon ingestion, and positive pressure ventilation [10, 11]. Three theories have been proposed to explain the formation of a pneumatocele: (a) pulmonary overinflation caused by check valve airway obstruction, (b) drainage of necrotic lung parenchyma with subsequent enlargement caused by check valve airway obstruction, and (c) focal air collection in pulmonary interstitial tissue caused by a direct communication between the pulmonary interstitium and parenchyma due to inflammation and necrosis of the airway wall [10]. A pneumatocele is usually transient and generally resolves spontaneously [54]; however, it is still associated with some complications. Specifically, a pneumatocele may be accompanied by infection [11, 54], and the development of the air-fluid level within the cyst may be observed in an infected pneumatocele [11]. Additionally, a pneumatocele may rupture through the pleural surface and result in pneumothorax [11, 54] or increase in size and compress the adjacent area and impair the cardiorespiratory system [11, 54].

A pneumatocele appears as a thin-walled, well-defined round air space in the lungs on radiographs and CT images [10, 11, 54, 55] (Fig. 19). It usually does not contain any fluid [55]. Pneumatoceles are transient and only exist for a limited amount of time (Fig. 19). At most, it takes weeks to months for a pneumatocele to disappear [54, 55].

## Diagnostic approach to abnormal air collection based on the location

### Decision trees

**Fig. 22:**
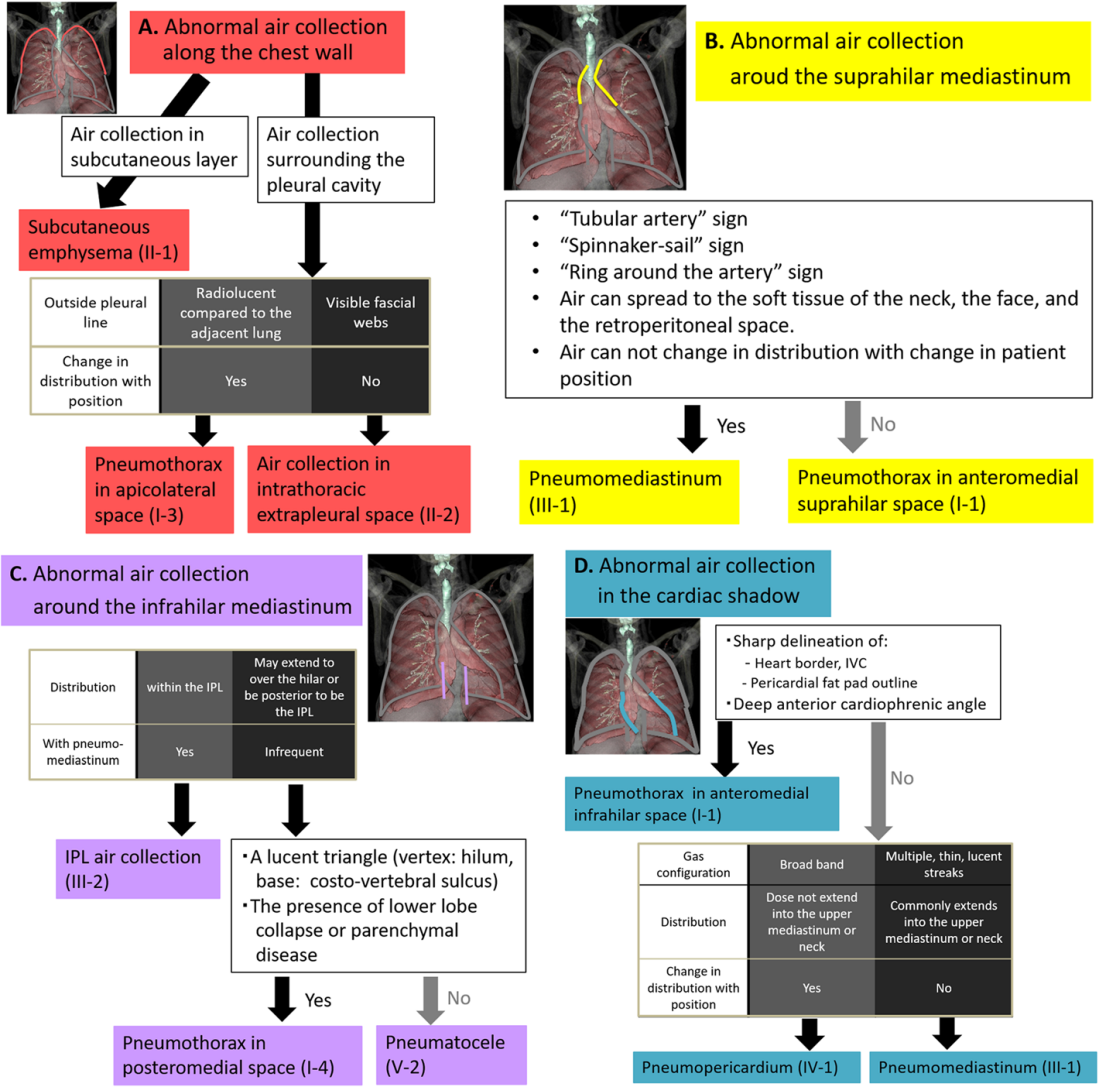
Decision trees based on the location of air collection. **a** Abnormal air collection along the chest wall. **b** Abnormal air collection around the suprahilar mediastinum. **c** Abnormal air collection around the infrahilar mediastinum. **d** Abnormal air collection in the cardiac shadow

We presented four decision trees for the location of air collections along the chest wall (Fig. 22a), around the suprahilar mediastinum (Fig. 22b), around the infrahilar mediastinum (Fig. 22c), and in the cardiac shadow (Fig. 22d). A differential diagnosis can be made when one of the four decision trees is selected depending on the location of air collection on a radiograph.

## Conclusion

A comprehensive understanding of three-dimensional chest anatomy based on CT enables the prediction of sites of air collection on radiographs in supine patients. The management of patients differs based on the location of the air collection; thus, radiologists should attempt to detect an abnormal air collection and accurately identify its location on radiographs.

## Data Availability

Not applicable.

## Abbreviations

ARDS: Acute respiratory distress syndrome
CT: Computed tomography
ICU: Intensive care unit
IPL: Inferior pulmonary ligament
IVC: Inferior vena cava
PIE: Pulmonary interstitial emphysema
RDS: Respiratory distress syndrome
SVC: Superior vena cava
